# Asteroid impact: the potential of astrocytes to modulate human neural networks within organoids

**DOI:** 10.3389/fnins.2023.1305921

**Published:** 2023-11-22

**Authors:** S. S. Lavekar, M. D. Patel, M. D. Montalvo-Parra, R. Krencik

**Affiliations:** Department of Neurosurgery, Center for Neuroregeneration, Houston Methodist Research Institute, Houston, TX, United States

**Keywords:** astrocytes, organoids, human pluripotent stem cells, synapses, extracellular matrix, microglia

## Abstract

Astrocytes are a vital cellular component of the central nervous system that impact neuronal function in both healthy and pathological states. This includes intercellular signals to neurons and non-neuronal cells during development, maturation, and aging that can modulate neural network formation, plasticity, and maintenance. Recently, human pluripotent stem cell-derived neural aggregate cultures, known as neurospheres or organoids, have emerged as improved experimental platforms for basic and pre-clinical neuroscience compared to traditional approaches. Here, we summarize the potential capability of using organoids to further understand the mechanistic role of astrocytes upon neural networks, including the production of extracellular matrix components and reactive signaling cues. Additionally, we discuss the application of organoid models to investigate the astrocyte-dependent aspects of neuropathological diseases and to test astrocyte-inspired technologies. We examine the shortcomings of organoid-based experimental platforms and plausible improvements made possible by cutting-edge neuroengineering technologies. These advancements are expected to enable the development of improved diagnostic strategies and high-throughput translational applications regarding neuroregeneration.

## Introduction

Astrocytes are abundant cells in the central nervous system (CNS) that serve myriad functions in maintaining the general homeostasis of neurons during healthy development, maturation, and aging. For example, astrocytes directly manipulate synaptic network formation and function through various signaling molecules (Saint-Martin and Goda, 2022; Farizatto and Baldwin, 2023), remodel the extracellular matrix (Tewari et al., 2022; Dzyubenko and Hermann, 2023), perform phagocytosis (Park and Chung, 2023), and regulate extracellular ions and neurotransmitters during neural activity (Andersen and Schousboe, 2023; Purushotham and Buskila, 2023). Beside neurons, astrocytes also conduct intercellular communication not only among themselves (Barber et al., 2021; Mazaud et al., 2021), but also to the neurovasculature (Diaz-Castro et al., 2023; Lia et al., 2023), other glia (e.g., oligodendrocytes and microglia; Liu et al., 2023), and peripheral immune cells (Han et al., 2021). In neuropathological conditions such as injury, inflammation, and neurodegenerative diseases, astrocyte signaling cues can become dysregulated and thus may be targets for therapeutic intervention (Brandebura et al., 2023; Lawrence et al., 2023; Patani et al., 2023; Qian et al., 2023). Currently, our understanding of astrocytes is based primarily on experimental rodent models. Because human and non-human primate astrocytes exhibit distinct features that are not recapitulated in rodents (Krencik et al., 2017a; Falcone and Martinez-Cerdeno, 2023), human-specific experimental models are also crucially needed.

**Figure 1:**
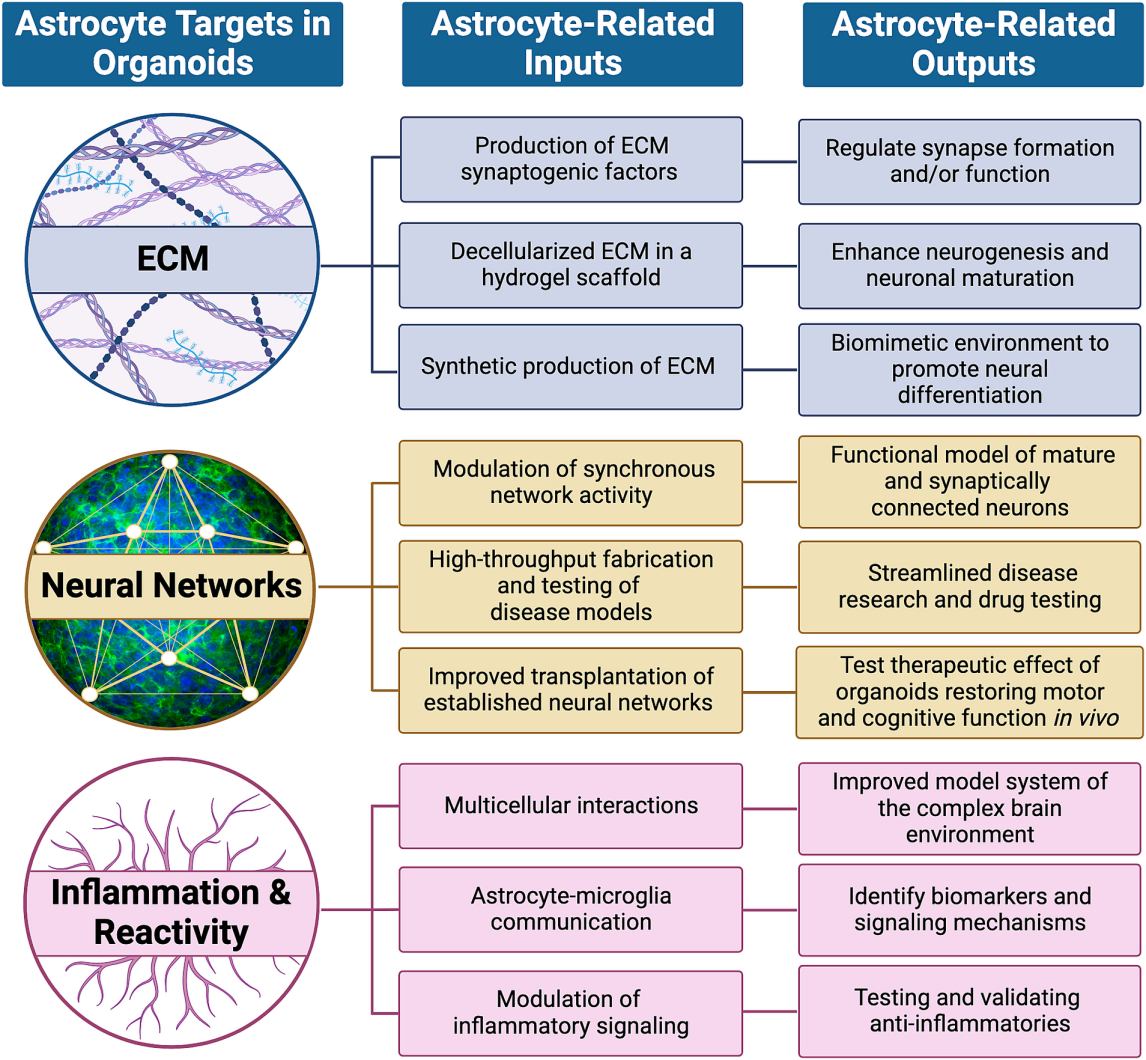
High priority targets within organoids that are expected to be modulated by astrocytes. Astrocytes dynamically regulate neuronal development and maturation in healthy and pathological conditions by influencing targets such as the extracellular matrix (ECM), neural networks and signaling with other non-neuronal cells to induce inflammation and/or reactivity. Their influence, or astrocyte-related inputs, can be subcategorized and potentially mimicked through synthetic means and lead to functional outcomes, or astrocyte-related outputs, as described.

For over a decade, astrocytes derived from human pluripotent stem cells (hPSCs) have been used as a cellular source to further understand basic neurobiology at the molecular and cellular levels, as well as for disease modeling, as we (Krencik et al., 2011, 2015; Krencik and Ullian, 2013; Patel et al., 2019) and others (Li and Shi, 2020; Bigarreau et al., 2022; Kumar et al., 2022; Jovanovic et al., 2023) have previously described. However, major caveats remain to be addressed in the traditional methods for astrocyte generation, including the inconsistent choice of differentiation timepoints to conduct studies, cell culture medium components, stress induced by cellular dissociation, use of synthetic substrates that can alter functionality, and lack of neurons and other cell types that contribute intercellular signals for astrocyte physiological maturity. Altogether, these insufficient culture conditions likely introduce artifacts that cause astrocytes in culture to not accurately represent those within the nervous system. To overcome these issues, there has been a constant optimization of differentiation protocols, design of multicellular coculture approaches, and implementation of physiological systems to improve hPSC-based astrocyte studies. Here, we focus on the use of maintaining hPSC-derived neural cultures as aggregate cell cultures to investigate astrocytes and their impact upon neuronal function. These aggregate cultures are originally known as neurospheres (Zhang et al., 2001), or spheroids, and more recently are commonly referred to as organoids and other nomenclature (Pasca et al., 2022; Jensen and Little, 2023). Organoids are composed of multicellular high density free-floating and self-assembling aggregate cultures that permit dynamic restructuring, maintenance of long-term intimate connectivity, and production of endogenous extracellular matrix (ECM) components. Numerous recent reviews have discussed the advantages and technological advancements of neural organoids (Andrews and Kriegstein, 2022; Wang et al., 2022; Yang et al., 2022; Wang L. et al., 2023; Zhou et al., 2023), yet few have focused on the impact of glia to modulate neuronal activity. Astrocytes can either be allowed to spontaneously arise as a small percentage of total cells over time within organoids (Lanjewar and Sloan, 2021), or be purposefully incorporated as a defined coculture approach for a more systematic system which we previously, tongue-in-cheek, named Asteroids (Krencik et al., 2017b; Cvetkovic et al., 2018). How would the presence of astrocytes alter baseline and disease-associated neuronal activity in organoids? How does the high density and stable environment of organoids alter maturity and function of astrocytes? Here, we summarize recent studies examining various components of organoids that will undoubtably be affected by the presence of astrocytes (i.e., inputs), and we provide perspectives on why the inclusion of astrocytes within neural organoids would provide a more accurate model of the human nervous system (i.e., outputs; Figure 1). This more complex version of organoids may improve their potential as a tool for accurate translational testing, such as transplantation therapy and drug testing to modulate neuroplasticity, neurodegeneration, and neuroregeneration.

## Impact of astrocyte-derived extracellular components in organoids

Astrocytes are an integral part of the CNS throughout the course of the life span. One of their important functions is producing and modulating ECM components that affect, among other things, neural activity. The ECM in the CNS can be generally subdivided into (a) basement membrane of the vasculature that plays a part in the blood brain barrier and is mainly composed of collagens, laminins, fibronectins, and heparin sulfate proteoglycans (HSPGs) among others (Banks et al., 2021; Knox et al., 2022); (b) the interstitial matrix that allows diffusion between cells and is composed of hyaluronan, chondroitin sulfate proteoglycans (CSPGs, including lectican core proteins aggrecan, versican, neurocan, and brevican with glycosaminoglycan [GAG] side chains), and tenascins among others (Shetty and Zanirati, 2020; Ghorbani and Yong, 2021; Tonnesen et al., 2023); and (c) perineuronal net (PNN) ECM that also consists of similar components, yet densely coats neurons to stabilize synapses and regulate neuroplasticity (Fawcett et al., 2019; Tewari et al., 2022; Dzyubenko and Hermann, 2023). Astrocytes produce many of these components as well as modulators such as matrix metalloproteinases (MMPs) and tissue inhibitors of metallo-preoteinases (TIMPs) that dynamically regulate the ECM at distinct stages of the lifespan and pathogenesis (Ulbrich et al., 2021; Allnoch et al., 2022). During astrocyte reactivity in response to traumatic injury, these components also play a role in the glial scar formation by inhibiting and/or supporting regeneration depending on the context such as severity of injury, time after injury, and distance to the epicenter (Escartin et al., 2021; Hemati-Gourabi et al., 2022; Tran et al., 2022). Finally, there have been many recent insights into another major component of extracellular signaling: astrocyte-derived non-structural matricellular ‘synaptogenic’ proteins that directly regulate synapse formation and/or function, such as thrombospondins, glypicans, Sparcl1, Pentraxin 3, etc. (Farizatto and Baldwin, 2023; Khaspekov and Frumkina, 2023). Altogether, and relevant to this review, questions remain whether these various types of ECM components and modulators are present within organoid culture models and how their absence or presence would alter neuronal function and thus interpretation of experimental data.

As ECM and other extracellular components derived from astrocytes play an integral role in synaptogenesis and/or function (Farhy-Tselnicker et al., 2017; Holt, 2023), an interesting question is whether they have been investigated within organoid models thus far. Compared to monolayer cultures of neurons and astrocytes, hPSC-derived neural organoids appear to be enriched in proteoglycans and cell adhesion proteins that are known to interact with the ECM (Simao et al., 2018; Cvetkovic et al., 2022; De Simone et al., 2023). On the other hand, monolayer cultures are enriched in collagens, suggesting that the use of matrices to attach monolayer cultures, or the stiffness on plates, may elicit a more fibrotic-like production of the ECM. Thus, it is likely that organoids will also produce different ECM components in the presence of astrocytes and/or when regionally specified. For example, specific ECM components that are present in the human retina have also been detected in retinal organoids, including fibronectin, versican, brevican, the retinal interphotoreceptor matrix protein IMPG1, and the transmembrane cell adhesion glycoprotein CD44. Blocking IMPG1 and CD44 with antibodies interfered with photoreceptor development (Felemban et al., 2018), demonstrating the potential functional consequence of modifying organoid matrix components either with exogenous application or by changing the cellular components. Because CD44 is produced by Müller glia in organoids (Eastlake et al., 2023) and by hPSC-based astrocytes (Krencik et al., 2011), the presence of astrocytes within organoids may affect functional maturation through their interactions with matrix components such as hyaluronic acid (HA). For example, direct digestion of HA with hyaluronidase in organoids, or treatment with purified HA, alters synapse density, while modulation of neuronal activity can be observed after dissociation of cells onto multielectrode arrays (MEAs; Wilson et al., 2020). However, to our knowledge, the contribution of astrocyte-derived ECM on neuronal function within organoids has not yet been rigorously investigated.

The addition of astrocyte-derived ECM into organoids via direct inclusion of astrocytes or exogenous application is an opportunity to better understand their functional contribution. One approach is to extract native ECM through a decellularization process, producing an ECM in a hydrogel scaffold. Then, researchers can subsequently use this scaffold to determine the effect upon organoid maturation and function. Decellularization typically consists of the chemical and enzymatic removal of cellular components while retaining the ECM. Decellularized human brain ECM, containing GAGs, collagens, proteoglycans, and various glycoproteins, has been discovered to enhance neurogenesis and physiological maturation of neurons within organoids as compared to Matrigel, which is a commonly used rodent tumor-derived basement membrane composed of collagens, laminins, and proteoglycans (Cho et al., 2021). Similarly, compared to Matrigel, ECM scaffolds sourced from porcine brains cause a morphological difference at early stages in ventricular-like structures and early appearance of neurons within organoids (Sood et al., 2019). Also, the use of fetal ECM-enriched constructs revealed an increased activity and downregulation of reactive astrocyte markers, although it also led to a decrease of GAGs and an increase of collagen compared to the native tissue (Simsa et al., 2021), indicating that the technique needs further optimization. Decellularization has also been performed using adult bovine neural retina retinal pigment epithelium (RPE; Dorgau et al., 2019). The addition of RPE-sourced ECM components increased the generation of photoreceptors as well as ribbon synapses and light responsiveness in retinal organoids. It remains to be known whether there is an optimal formulation of ECM components, either cell-derived or synthetically generated, to influence neural organoid function and whether an astrocyte-specific ECM formulation would have additional benefits compared to other cell types.

Astrocyte-derived, or astrocyte-inspired, ECM compositions can also lead to distinct autonomous and/or non-autonomous impacts. For example, protoplasmic astrocytes derived from mouse embryonic stem cells in monolayers produce elevated levels of permissive ECM components, such as laminin α5 and γ1, in contrast to fibrous astrocytes that produce inhibitory ECM components, such as spondin-1, that limit axonal growth capacity (Thompson et al., 2017). Primary rat astrocyte-derived ECM has been validated to protect against foreign body response that typically occurs due to microelectrode implants within brain tissue. In comparison to uncoated electrodes, astrocyte-derived ECM coating increased protection against macrophage activation and decreased astrogliosis (Oakes et al., 2018). Testing of distinct combinatorial formulations of three-dimensional (3D) ECM hydrogels with collagen, HA, and Matrigel revealed different degrees of reactivities of human fetal-derived astrocytes (Placone et al., 2015). In addition to distinct composition of ECM components, physical properties such as stiffness can also elicit various amounts of cellular reactivity. For example, in a 3D hydrogel primary rat astrocyte culture, comparison of soft, medium, and stiff matrices revealed a differential response in cell shape and glial fibrillary acidic protein (GFAP) levels (Hu et al., 2021). In regards to organoids, because incorporation of defined scaffolds and matrices have already demonstrated an impact on structure (Lancaster et al., 2017; Muñiz et al., 2023), it is highly expected that astrocyte-derived ECM and/or astrocyte-inspired synthetic ECM would significantly affect long-term function of astrocytes and other constituents within organoids that will lead to the development of a more useful platform for *in vitro* drug analyses and high throughput studies.

Dysregulation of astrocyte ECM is also expected to be a phenotypic consequence of disease states that can subsequently impact neuronal function, similar to what we previously observed with a model of Costello syndrome that displayed a dysregulated ECM and increase in synapse density using monolayer cultures (Krencik et al., 2015) and Asteroids (Krencik et al., 2015; Cvetkovic et al., 2018). In a neural organoid model of amyotrophic lateral sclerosis/frontotemporal dementia, single cell sequencing revealed a large number of differentially expressed genes in astrocytes including those involved in ECM remodeling and synaptic plasticity (Szebényi et al., 2021). In an organoid model of neurotoxicity using the cancer therapy drug Vincristine, dose-dependent toxicity of neurons and astrocytes was detected as well as a downregulation of ECM transcripts (Liu et al., 2019). Altogether, organoid models have demonstrated validity to explore the effect of drugs and genetic manipulations on changes in the ECM, and vice versa, albeit the exploration of astrocyte-specific contributions to these phenotypes has been limited. For example, there is still a lack of reproducible injury or inflammation-based organoid models to study the effect of glial scar components. Further, despite organoid studies that observe ECM changes in distinct contexts (Jabali et al., 2022; Pipicelli et al., 2023), the vast majority of approaches utilize immature organoids that do not contain a significant amount of astrocytes or mature neural network activity to dissect how a dysregulated ECM can lead to dysfunction in the mature and aged brain. Thus, there is a clear need for improved organoid models and technologies to understand the role of astrocyte-derived ECM in normal and diseased states, as well as testing ECM-based formulations for neuroprotection and/or neuroregeneration of mature neural networks.

## Astrocyte coordination of neural network activity within organoids?

Neural networks in the CNS are interconnective systems of neurons that integrate and process information for signal transmission. These networks are complex and diverse; their make-up consists of various neuron types with regards to function and morphology. For example, networks can be composed of different ratios of excitatory and inhibitory subtypes of neurons that secrete different types of neurotransmitters, and they can be distinct depending on the brain region (Hanganu-Opatz et al., 2021; Xing et al., 2023). Neural networks are often defined by synchronous and oscillatory neuron activity that can vary in brain states and are dependent on synaptic connectivity within or across regions (Whittington et al., 2018). Additionally, integrated as part of the neural network is the diversity of supporting glial cells that contribute to the development and maturation of neural networks (Knowles et al., 2022; Pathak and Sriram, 2023; Whitelaw et al., 2023). Due to their extensive morphological branching, a single astrocyte may contribute to the maintenance of thousands of synapses in a neural network (Stogsdill et al., 2023). How do astrocytes specifically communicate with networks? In addition to their well-known regulation of extracellular potassium and glutamate, a diverse milieu of neurotransmitters activate receptors on participating astrocytes and cause signaling pathway activation, such as increased intracellular calcium that may lead to secretion of active substances (a.k.a., gliotransmitters) that then feedback into the network and alter network activity (Oliveira and Araque, 2022). For example, an astrocyte subpopulation in the rodent brain has been recently identified that secretes glutamate in response to stimulation (de Ceglia et al., 2023).

Currently, the formation of neural networks is enabled by the production and maintenance of organoids over a long period of time; numerous modifications and different technologies to do so have ultimately confounded comparison across different studies. However, the vast majority of fabrication techniques still follow a traditional approach that involves permitting hPSC-derived neural progenitors to self-aggregate and differentiate in a neural-supportive media with the addition of growth factors such as brain-derived neurotropic factor (Zhang et al., 2001). Over time, organoids further mature and are composed of a mixture of neural progenitor cells, neuron- and astrocyte-subtypes, as well as non-neural components, depending on the efficiency of neural induction. More recent modifications of this approach include the use of engineering techniques (e.g., sphere-forming molds, 3D printing, bioreactors, microfluidic systems, and biomaterials) to control size, improve reproducibility, and incorporate microenvironments more similar to *in vivo* conditions (Kratochvil et al., 2019). Similar to monolayer approaches, organoids can also be specified into region-specific progenitor cells to generate subtypes of neural networks. For example, treatment with molecules to activate WNT and Sonic Hedgehog pathways can generate organoids with midbrain characteristics that contain tyrosine hydroxylase positive dopaminergic neurons for Parkinson’s disease modeling (Monzel et al., 2017; Renner et al., 2020). More recently, direct differentiation techniques have been incorporated to accelerate maturation into specific cell types. For example, we used transcription activator -like effector nuclease (TALEN) genetic engineering to directly differentiate neural cells via the use of a doxycycline inducible transcription factors, neurogenin 2 (NGN2) for neurons and SRY-box transcription factor 9 (*Sox9*) and nuclear factor 1 A (NFIA) for astrocytes. Thus, direct differentiation helped to rapidly produce postmitotic neurons and astrocytes, which were used to form all-inducible organoids (a.k.a., Asteroids) in specific cellular ratios that do not change over time (Cvetkovic et al., 2018). In either case, neural organoids have potential to eventually mature into a neural network of neurons communicating via functionally active synapses. The resultant neural networks can be used to discover and investigate factors that modulate network activity and to model disease processes that are known to occur within the human brain.

A key standard in defining neural networks within organoids is the presence of the synapses that ultimately contribute to the formation of network activity. These can be defined within organoids using several approaches. Immunostaining for synaptic markers is one of the standard methods to verify the presence of synaptic structures that contribute to the formation of network activity. Several studies have observed the presence of presynaptic markers, including synaptophysin, synapsin, and VGLUT1, as organoids mature (Thomas et al., 2017; Sakaguchi et al., 2019; Trujillo et al., 2019). The additional detection of colocalized pre-synaptic markers with post-synaptic markers, including PSD-95 and Homer, further indicates the presence of a synapse with increased rigor (Pasca et al., 2015; Monzel et al., 2017; Cvetkovic et al., 2018; Renner et al., 2020). Within Asteroids, we observed a close proximity of synapses with astrocyte processes (Cvetkovic et al., 2022). Despite the presence of synapses throughout organoids, there is no guarantee that they have functional transmission. As a result, functional assays should be used to verify simultaneous activation of spatially distinct neurons in culture. For example, neuron monocultures exhibit increases of action potentials and synchronicity across several electrodes on MEAs during prolonged culture, and this phenomenon had enabled testing of epilepsy-related drugs (Odawara et al., 2016). Similarly, when we plated mature organoids containing astrocytes upon MEAs, synchronous activity was detected across several electrodes that was subsequently blocked by the addition of the AMPA/kainate receptor antagonist, CNQX, suggesting that synaptic activity mediated synchronous activity (Cvetkovic et al., 2018). In paradigms that include inhibitory neurons within organoids, the addition of bicuculline to block GABAergic transmission increases synchronous events (Trujillo et al., 2019). In the case of region-specific midbrain organoids, the addition of quinpirole, an agonist of the inhibitory D2 and D3 receptor, reduced synaptic neuron activity, suggesting the presence of synaptically connected dopaminergic networks (Monzel et al., 2017). Ideally, a combination of these different approaches should be combined to conclusively verify the presence of active synapses within organoids.

Another common approach to measure synchronous network activity is live calcium imaging using fluorescent calcium sensors and genetically encoded calcium indicators (GECIs). In one study, although synchronous calcium activity could not be detected in organoids after 104 days in culture, dissociation and reformation of clusters over time led to synchronized network calcium activity that was increased by applying glutamate and blocked by GABA and CNQX (Sakaguchi et al., 2019). However, synchronous calcium transients have been observed in other laboratories within organoids (Renner et al., 2020) without the need for dissociation, suggesting differences in culture conditions and protocols can have a dramatic effect on network activity. To our knowledge, whether the presence of astrocytes (in normal or disease-relevant states) modulates calcium-based measurements of network activity in organoids has yet to be investigated, though data using monolayer culture studies support that astrocytes are likely to have a positive influence. For example, neuron monolayer cultures containing astrocytes exhibited synchronous calcium activity after 15 weeks of culture. In contrast, adding cytosine-beta-D-arabinofuranoside to deplete proliferating progenitors reduced glia and resulted in a complete lack of spontaneous network activity (Klapper et al., 2019). We expect it will also be informative to use a GECI approach specifically in astrocytes to investigate if, and when, astrocytes become a functional part of tripartite synapse within organoids.

Because fluorescent calcium sensors and rigid MEAs have drawbacks (e.g., the inability to measure activity beyond a small portion of the organoid surface), engineers have developed arrays that envelop and encase organoids throughout the entire surface of the structure. Various designs have been developed, including shell-like designs that encase organoids in a transparent sphere allowing for simultaneous microscopy (Huang et al., 2022) and rollable electrode arrays that are able to fit various sized organoids (Kalmykov et al., 2021). These devices have shown promise so far with regards to a high signal-to-noise ratio and ease in analyzing changes in neuron activity when adding neuroactive compounds such as glutamate. Alternatively, for integration of electrodes throughout organoids, a flexible and stretchable electrode-laden mesh can be seeded with human stem cells and allowed to self-organize, producing an incorporated mesh of electrodes capable of both measurement and stimulation of the cells in an approach termed “cyborg organoids” (Li et al., 2019). It remains to be seen if the caveats of using these more complex methods for sensing neural activity are advantageous over the more user-friendly current state of the art. Regardless, it will be important to devise, optimize, and validate innovative methods for long-term, high-resolution, and high-throughput sensors for increased accuracy, reproducibility, and to enhance scientific rigor in the field.

In parallel to detecting neural networks, methods are needed to modulate activity in organoids. One promising method for modulation and to model clinically relevant functional neurosurgery is electrical stimulation. *In vivo* studies have discovered that prolonged electrical stimulation can promote neuronal axon growth and integration within the spinal cord to restore motor function in monkeys and rats (Alam et al., 2017; Barra et al., 2022). If electrical stimulation of neurons *in vivo* shows promise in re-establishing neural networks to restore motor function, then it stands to reason that electrical stimulation of organoids may help promote neural network development and provide a novel system to test stimulation parameters. Investigators are currently exploring the application of direct electrical stimulation to organoids through tools such as the Utah Array to penetrate and stimulate organoids (Morales Pantoja et al., 2023). Unfortunately, a major caveat with this approach is that chronic electrical stimulation is technically challenging because the immature organoids are still developing and experience high movement within bioreactors or shaking flasks. To address this limitation, an electric field application has been alternatively used for stimulation, and this approach has resulted in increased axon growth and synapse density in both the mouse brain (Grossman et al., 2017) and organoid-like aggregate cultures (Meng et al., 2020), suggesting its capability to induce a more robust neural network. An alternative stimulation strategy that is commonly used for research, but not currently feasible for clinical translation, is genetically encoded optogenetic stimulation to target a specific population of cells engineered with light-sensitive ion channels. Similar to electrical stimulation, optogenetic stimulation of rats with cervical spinal cord injury improved forelimb recovery that was mediated by an increase in axonal growth and glutamatergic and GABAergic synapses (Mondello et al., 2023). Optogenetic studies in organoids have been more limited mainly due to the challenges of genetic engineering and long-term light stimulation (Morales Pantoja et al., 2023), though these studies have indeed been conducted (Osaki et al., 2018; Andersen et al., 2020; Revah et al., 2022; Harley et al., 2023). Our lab has confirmed that optogenetic stimulation of neurons in organoids is feasible and permits the detection of astrocyte-dependent changes in activity during coculture (Cvetkovic et al., 2022). This is an emerging field with promise, yet there is a clear need for optimized technologies to reproducibly stimulate neural organoids for neuromodulation studies. An important future direction in this field would be to generate closed-loop feedback with organoid-computer interfaces to modulate activity in real time as has been recently demonstrated in a simulation of the game “Pong” (Kagan et al., 2022).

With improved organoid fabrication techniques as well as unique ways to characterize the viability and physiology of neural networks, researchers can take a further step to model neuropathological conditions that involve synaptic networks. Neurexin 1 (NRXN1) deletions are linked to increased risk in schizophrenia. Along with cell-specific transcriptional changes, organoids with NRXN1 deletions displayed decreased synchronized calcium burst activity at 4–5 months of culture compared to control organoids (Sebastian et al., 2023). Network oscillations have also been found to be altered in organoids subject to different pressure shockwaves modeling primary blast injury. Organoids exposed to high-pressure shockwaves had significant reduction in synchronous neuron firing as measured by MEAs (Silvosa et al., 2022). On the other hand, diseases featuring hyperactive neural networks have also been modeled in organoids. For example, neural organoids with MECP2 mutations, which are associated with Rett syndrome (a disease that commonly features epileptic symptoms), led to an increase in excitatory synaptic puncta, an increase in synchronized calcium transients, and increase in burst spike activity. Further, local field potential recordings of mutant organoids revealed a lack of low frequency oscillations that were instead replaced by epileptiform-appearing spike activity with high frequency oscillations. This epileptic model demonstrates that treatment of an anti-epileptic drug, sodium valproate, can reduce spike activity in mutant organoids. Additionally, treatment with a target inhibitor of TP53, which has been linked to Rett syndrome, reduced spike frequency and high frequency oscillations of local field potential measurements (Samarasinghe et al., 2021). Similarly, organoid models of Angelman’s syndrome with UBE3A have disruptions in ubiquitin-mediated degradation of Ca^2+^ and voltage dependent big potassium (BK) channels that result in hyperactivity and rescued with the BK antagonist paxillin (Sun et al., 2019). In cancer model, neural organoids in coculture with glioblastoma (GBM) cells exhibit increased functional burst activity as measured on MEAs with increased synapse puncta density. Interestingly, these overactive neural networks may result in increased growth of the GBM cells through thrombospondin-1 signaling, supporting the idea that neural network hyperactivity positively affects GBM growth (Krishna et al., 2023). Altogether, it is becoming clear that organoid-approaches are feasible and reliable models to investigate neuropathologies that effect neural activity as an alternative to monolayer cultures and animal models, though there remains distinct advantages and caveats to each approach.

Can astrocytes enhance the therapeutic ability of transplanted organoids to rescue diseased or structurally impaired neural networks? hPSC-derived dissociated astrocytes have been engrafted into animal models by our lab (Krencik et al., 2011) and others (Hastings et al., 2022). However, to the best of our knowledge, astrocytes have not been specifically incorporated into organoids to test this question. Still, transplantation of organoids is emerging as a feasible alternative approach instead of the injection of dissociated cells. Midbrain specific organoids containing dopaminergic neurons have been transplanted into the striatum of Parkinson’s disease mouse models. The neurons then formed synaptic connections with host tissue and improved motor function in three motor tests (i.e., apomorphine-induced rotation, open field, and rotarod testing; Zheng et al., 2023). Alternatively, cortical organoids have been engrafted into the S1 region of a mouse cortex and exhibit connectivity via sensory whisker stimulation-induced activity. Further, optogenetic stimulation of organoids was discovered to drive reward-seeking behavior (Revah et al., 2022). In regard to the visual system, organoids have been transplanted into large injury cavities of the visual cortex and respond to orientation selective visual stimulation (Jgamadze et al., 2023). As a final example, organoids that were transplanted into a stroke-mediated injury model increased repair of the infarcted core, potentially by means of increased differentiation into glial cells compared to injection of single-cell suspension. Behavioral tests were also performed, indicating improved performance on all sensorimotor function (Cao et al., 2023). Though these studies demonstrate efficacy, the advancements and novelty of using organoids over previous transplantation methods using fetal tissue aggregates and dissociated hPSC-derived cells are not clearly apparent and this approach needs major advancement before it can realistically be translated to the clinic. In summary, with the discussed pioneering advancements of both the manipulation, measurement, and utilization of neural networks in organoids, this exciting field of basic and translational research is primed to rapidly expand. While we postulate that astrocytes or astrocyte-inspired biomaterials will be an important addition to model neural networks, other cell types will also be needed for accurate experimental models.

## Beyond the asteroid: multicellular crosstalk coordinates inflammatory signaling

Besides direct astrocyte-neuron interactions, there are multiple other cell types that may directly or indirectly modify this relationship. Here, we will focus on the role of microglia as they have been the subject of numerous neural organoid studies. Astrocytes and microglia remain in constant communication and coordinate their functions with crosstalk and feedback signaling cycles (Linnerbauer et al., 2020; Matejuk and Ransohoff, 2020; Vainchtein and Molofsky, 2020). In brief, microglia are a self-replicating resident immune component within the CNS that originates from non-neural lineages (Lazarov et al., 2023). Like astrocytes, microglia have numerous functional roles and have traditionally been characterized as either being in resting or activated states based on morphology and biomarker expression. However, more complex and dynamic sub-classifications have recently been proposed based on specific contexts, including sex, age, and environmental cues (Paolicelli et al., 2022). Microglia express a wide array of receptors, including those for neurotransmitters and cytokines, which enables them to respond rapidly to a plethora of environmental changes by releasing signaling molecules (e.g., inflammatory cytokines and neurotrophic factors). Further, they contribute to neuroplasticity through synaptic refinement, synaptic pruning, and ECM remodeling (Sancho et al., 2021; Wong and Favuzzi, 2023). Altogether, these roles have been implicated as a cause and/or contributor to neurodegeneration and other neurological diseases (Scott-Hewitt et al., 2023). Similar to astrocytes, microglia have a long and diverse history of generation from hPSCs in order to investigate their baseline and disease-associated functions both as monolayer cultures and within organoids (Cakir et al., 2022; Garcia-Epelboim and Christian, 2023; Maksour and Ooi, 2023; Warden et al., 2023; Wenzel et al., 2023; Zhang et al., 2023). In this section, we will review recent research focused on microglia within organoid cultures and contemplate how their presence in organoids may help establish a better model to understand astrocyte-neuron–microglia interactions within different disease relevant conditions.

One of the main roadblocks to systematic inclusion of microglia, and multiple additional cell types, within organoids is the lack of standardization in neural organoid production. Various adjustments to protocols have been implicated to increase reproducibility and complexity of the resultant cellular population (Velasco et al., 2019; Shi et al., 2020; Sidhaye and Knoblich, 2021; Rosebrock et al., 2022; Sozzi et al., 2022). To avoid obstacles such as uncontrolled size, merging within culture conditions, and anoxia, there have been innovative solutions proposed from the field of microfluidics (Zhu et al., 2023), acoustofluidics (Ao et al., 2021a; Cai et al., 2021), and scaffolding engineering (Rothenbucher et al., 2021). Likewise, there are various strategies to generate microglia from hPSCs by either permitting them to spontaneously arise within organoids or to purposely incorporate them. For example, if neuroectoderm specification is not completely efficient during neural organoid differentiation, mesoderm-derived progenitors can give rise to microglia within organoids. These microglia express microglia biomarkers and respond to lipopolysaccharide (LPS) treatment by releasing inflammatory cytokines (Ormel et al., 2018). Alternatively, neural progenitors can be generated with efficient neural induction and, before organoid production, cocultured with specific numbers of microglia that are able to prune synapses and become inflamed after viral infection (Xu et al., 2021). A similar strategy has been used to incorporate microglia in midbrain-specified organoids (Sabate-Soler et al., 2022) and retinal organoids (Usui-Ouchi et al., 2023). In a different approach, a tubular organoid-on-a-chip device has been used that forces microglia to migrate into organoids to study neuroinflammation while ensuring oxygen perfusion, thereby minimizing necrosis and hypoxia (Ao et al., 2021b). In addition to optimizing the strategy for including microglia into organoids, it is important to also consider the purity and age of the microglia as this likely also alters their function and communication with other cell types. One promising approach to rapidly produce synchronous microglia is direct differentiation directly from hPSCs using a single transcription factor (Sonn et al., 2022) or a combination of factors (Drager et al., 2022), though burdens still remain in these protocols as they require exogenous treatments of various exogenous factors to promote development and maturation. Altogether, regardless of the approach, it will be critical that studies accurately report their methods in detail in order to ensure reproducibility among various laboratories.

Whether the same intercellular inflammatory signaling observed *in vivo* also occurs between hPSC-derived microglia and astrocytes within organoids remains to be rigorously explored. In monolayer cultures, tri-cultures of neurons, astrocytes, and microglia have been used to model Alzheimer’s disease (Bassil et al., 2021; Guttikonda et al., 2021). Thus, it is expected that organoid cultures can effectively be utilized to examine astrocyte-microglia interactions in models of aged and/or diseased inflammatory conditions. Additionally, transplantation of organoids directly into the CNS may be an opportunity to study microglia-astrocyte interactions within a more complex microenvironment. For example, organoids that have been seeded with CD43+ erythromyeloid progenitors survive longer and mature better when transplanted into the mouse cortex, potentially due to the absence of ligands *in vitro* that activate the CSF-1 receptor, vascularization of organoids, and appearance of mature astrocytes over time (Schafer et al., 2023). Microglia within these organoid transplants exhibit morphological differences in response to systemic intraperitoneal injection of LPS as well as in the context of cells derived from idiopathic autism spectrum disorder. Thus, incorporating more complex cellular diversity within organoids or incorporating organoids into a more complex microenvironment will undoubtedly increase their relevance as a model for disease states and for cellular transplantation therapy.

## Conclusion and future perspectives

**Figure 2:**
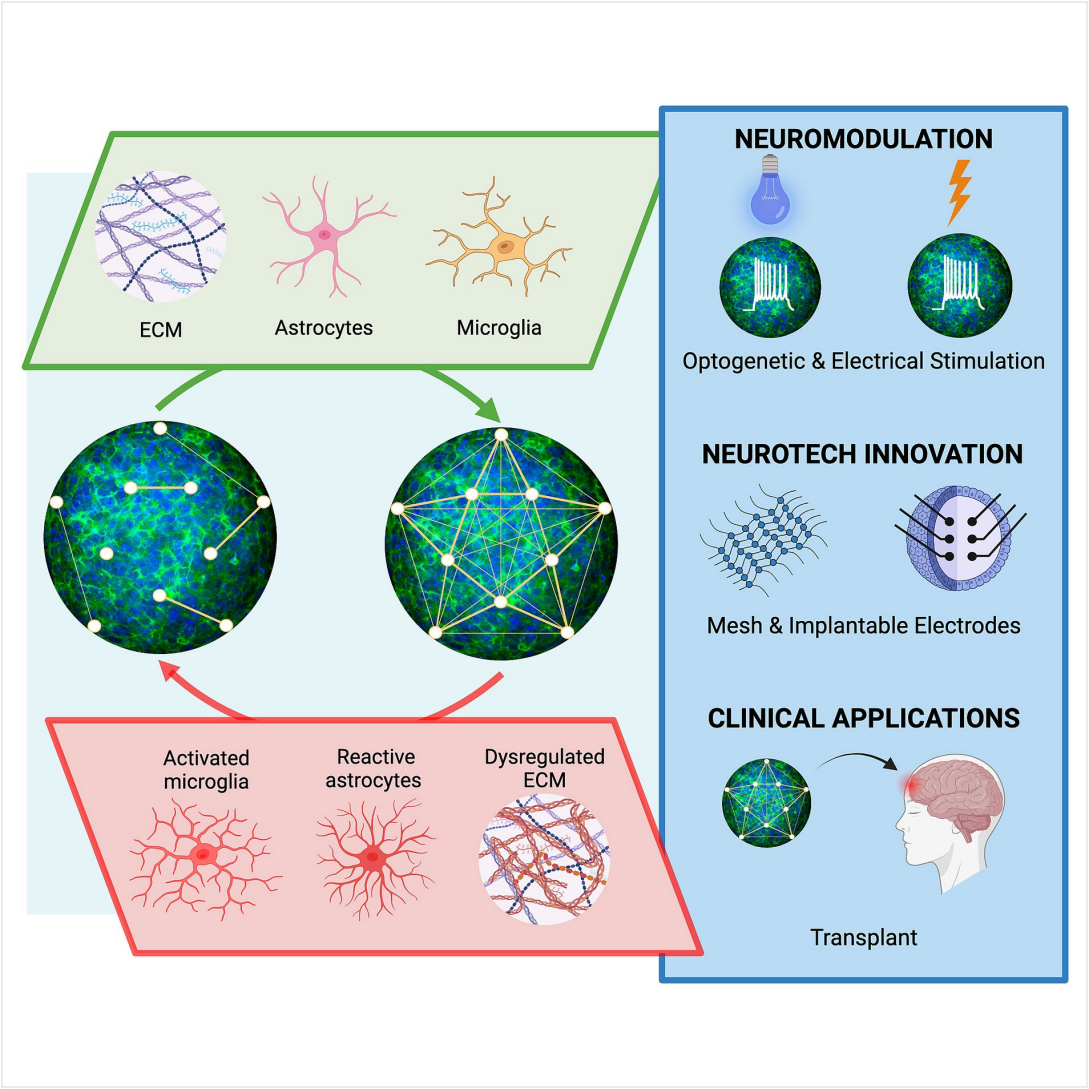
Astrocyte-dependent influences on human neural networks and emerging technological advancements. Neural connectivity in organoids may be strengthened (top), or weakened (bottom), through astrocyte-derived components including extracellular matrix (ECM), synaptogenic factors, gliotransmitters, and cytokines that affect the crosstalk between cells such as microglia. Emerging technologies, including those that modulate and sense neural activity, will be needed to investigate the influence of astrocytes on network function in organoids. Further improvements in establishing mature synaptic networks in organoids will advance support of established scientific findings and more clinically relevant strategies, high throughput drug screens and cell replacement therapy approaches.

Here, we have summarized major astrocyte-related components and technological innovations that we expect would either enhance or disrupt organoid function as defined by various experimental readouts (Figure 2). We further discuss the expected resultant consequences of organoid development, maturation, and function, as well as the relevance of organoids in modeling the complex human brain. However, major caveats remain, such as low reproducibility, immaturity, and insufficient cellular complexity in ratios similar to those within the CNS. Further, optimizations of the organoid model system and technological advancements are emerging, including computations tools that are needed to decipher the complex molecular networks and mechanisms underlying the myriad of astrocytic functions. For example, high resolution serial electron microscopy and computer vision models have recently been employed to dissect mouse astrocytic nanoarchitecture that is difficult to visualize with traditional methodology (Salmon et al., 2023). Combinations of high resolution microscopy (Torres-Ceja and Olsen, 2022) and advances in expansion microscopy (Sarkar et al., 2022) using organoids will undoubtably answer further questions with respect to the chemistry of astrocytes with synaptic clusters. Although organoid transplants show promise in integrating and repairing damaged neural networks, there exist substantial limitations in controlling maturation, connectivity, and viability, though incorporating biomaterials or genetic approaches to enhance vascularization into organoids is promising (Wang M. et al., 2023). The inclusion of astrocytes would likely enhance the ability of organoids to interface with the vascular through contact with donor astrocyte endfeet. With the addition of microglia in transplants, there is potential to take advantage of inflammatory reactions of glia as theranostic tools or cellular factories to produce genetically engineered anti-inflammatory molecules. Altogether, there is hope that traditional low efficient methodologies for neural organoid generation and experimentation will become extinct due to the impact of more systematic bioengineering-inspired neural organoids such as Asteroids and future innovations.

## Author contributions

SL: Writing – original draft, Writing – review & editing. MP: Writing – original draft, Writing – review & editing. MM-P: Writing – original draft, Writing – review & editing. RK: Writing – original draft, Writing – review & editing.
